# Supplementary material for: Risk factors for early childhood disability in Bangladesh: Evidence from Multiple Indicator Cluster Survey 2019
Source: PLoS One. 2021 Nov 4;16(11):e0259532. doi: 10.1371/journal.pone.0259532 (PMC8568190; doi:10.1371/journal.pone.0259532)
Supplement: S1 Table — (DOCX) [file pone.0259532.s001.docx]

**S1 Table. Table of the outcome and independent variables clearly describing their potential nature and values and codes**

| **Variable Name** | **Potential nature** | **Codes** | **Frequency (%)** |
| --- | --- | --- | --- |
| **Outcome Variable** | | |  |
| Disability Type | Ordinal | 0 = No disability | 13680 (97.2%) |
|  |  | 1 = At Least one disability | 285 (2%) |
|  |  | 2 = More disability | 107 (0.8%) |
| **Independent variable** | | |  |
| **Child level variables** | | |  |
| Age | Categorical | 2 = 2 years | 4610 (32.8%) |
|  |  | 3 = 3 years | 4832 (34.3%) |
|  |  | 4 = 4 years | 4630 (32.9%) |
| Sex | Categorical | 1 = Male | 7321 (52.0%) |
|  |  | 2 = Female | 6751 (48.0%) |
| Early childhood education attendance | Categorical | 1 = Attending | 1787 (12.7%) |
|  |  | 2 = Not attending | 7675 (54.5%) |
|  |  | 9 = Missing | 4610 (32.8%) |
| Measures Underweight -2 SD | Categorical | 00 = Not underweight | 10221 (72.6%) |
|  |  | 100.00 = Underweight | 3371 (24.0%) |
|  |  | 900.00 = Missing | 479 (3.4%) |
| Measure Stunt- 2 SD | Categorical | 00 = Not stunted | 9310 (66.2%) |
|  |  | 100.00 = Stunted | 4063 (28.9%) |
|  |  | 900.00 = Missing | 699 (5.0%) |
| Birth order | Categorical | 1 = First born | 11425 (81.2%) |
|  |  | 2 = Second born | 2287 (16.3%) |
|  |  | 3 = Third up to last | 59 (0.4%) |
|  |  | 9=Missing | 300 (2.1%) |
| **Family level variables** | | |  |
| Mother's Age | Ordinal | 1 = 15-19 years | 430 (3.1%) |
|  |  | 2 = 20-24 years | 3033 (21.6%) |
|  |  | 3 = 25-29 years | 3384 (24.0%) |
|  |  | 4 = 30-34 years | 2709 (19.3%) |
|  |  | 5 = 35-39 years | 1353 (9.6%) |
|  |  | 6 = 40-44 years | 401 (2.8%) |
|  |  | 7 = 45-49 years | 162 (1.2%) |
|  |  | 9=Missing | 2600 (18.5%) |
| Mother's education | Ordinal | 00 = Pre-primary or none | 1727 (12.3%) |
|  |  | 1 = Primary | 3409 (24.2%) |
|  |  | 2 = Secondary | 6845 (48.6%) |
|  |  | 3 = Higher secondary+ | 2090 (14.9%) |
| Mother's functional difficulties | Categorical | 1 = Has functional difficulty | 223 (1.6%) |
|  |  | 2 = Has no functional difficulty | 13581 (96.5%) |
|  |  | 9 = No information | 269 (1.9%) |
| Mother's age at birth | Ordinal | 1 = <20 | 7082 (50.3%) |
|  |  | 2 = 20-34 | 6649 (47.2%) |
|  |  | 3 = 35+ | 40 (0.3%) |
|  |  | 9=Missing | 300 (2.1)% |
| Estimation of overall happiness | Ordinal | 1 = Very happy | 2943 (20.9%) |
|  |  | 2 = Somewhat happy | 6899 (49.0%) |
|  |  | 3 = Neither happy or unhappy | 1211 (8.6%) |
|  |  | 4 = Somewhat unhappy | 228 (1.6%) |
|  |  | 5 = Very unhappy | 191 (1.4%) |
|  |  | 9=Missing | 2600 (18.5%) |
| **Community level variables** | | |  |
| Area | Categorical | 1 = Urban | 2949 (21.0%) |
|  |  | 2 = Rural | 11122 (79.0%) |
| Division | Categorical | 10 = Barishal | 809 (5.7%) |
|  |  | 20 = Chattogram | 3092 (22.0%) |
|  |  | 30 = Dhaka | 3317 (23.6%) |
|  |  | 40 = Khulna | 1468 (10.4%) |
|  |  | 45 = Mymenshing | 1039 (7.4%) |
|  |  | 50 = Rajshahi | 1700 (12.1%) |
|  |  | 55 = Rangpur | 1511 (10.7%) |
|  |  | 60 = Sylhet | 1135 (8.1%) |
| Antenatal care visit | Categorical | 1 = 1-4 visits to any provider | 137 (1.0%) |
|  |  | 2 = 5 or more visits to any provider | 59 (0.4%) |
|  |  | 9=Missing | 13875 (98.6%) |
| Delivery at home | Categorical | 1 = Yes | 112 (0.8%) |
|  |  | 2 = No | 139 (1.0%) |
|  |  | 9=Missing | 13820 (98.2%) |
| Ever used internet | Categorical | 1 = Yes | 1524 (10.8%) |
|  |  | 2 = No | 9852 (70%) |
|  |  | 3 = No response | 2691 (19.1%) |
| Mobile phone owner | Categorical | 1 = Yes | 9147 (65%) |
|  |  | 2 = No | 2323 (16.5%) |
|  |  | 3 = No response | 2600 (18.5%) |
