# Supplementary material for: Risk factors for early childhood disability in Bangladesh: Evidence from Multiple Indicator Cluster Survey 2019
Source: PLoS One. 2021 Nov 4;16(11):e0259532. doi: 10.1371/journal.pone.0259532 (PMC8568190; doi:10.1371/journal.pone.0259532)
Supplement: S2 Table — (DOCX) [file pone.0259532.s002.docx]

**S2 Table. The exact items assessing the different types of disabilities**

| **Disability type** | **Question** | **Codes** |
| --- | --- | --- |
| Seeing | Child has difficulty seeing (Code 3,4) | No difficulty ........................................................1  Some difficulty.....................................................2  A lot of difficulty .................................................3  Cannot see at all...................................................4 |
| Hearing | Child has difficulty hearing sounds  like people voices or music (Code 3,4) | No difficulty ........................................................1  Some difficulty.....................................................2  A lot of difficulty .................................................3  Cannot hear at all..................................................4 |
| Walking | Without using equipment or assistance child has difficulty walking (Code 3,4) | Some difficulty.....................................................2  A lot of difficulty .................................................3  Cannot walk at all................................................4 |
|  | When using equipment or assistance child has difficulty walking (Code 3,4) | No difficulty ........................................................1  Some difficulty.....................................................2  A lot of difficulty .................................................3  Cannot walk at all.................................................4 |
|  | Compared with children of the same age, child has difficulty walking (Code 3,4) | No difficulty ........................................................1  Some difficulty.....................................................2  A lot of difficulty .................................................3  Cannot walk at all.................................................4 |
| Fine motor | Compared with children of the same age, child has difficulty picking up small  objects with his/her hand (Code 3,4) | No difficulty ........................................................1  Some difficulty.....................................................2  A lot of difficulty .................................................3  Cannot pick up at all............................................ 4 |
| Communication | Child has difficulty understanding  parent/caretaker (Code 3,4) | No difficulty ........................................................1  Some difficulty.....................................................2  A lot of difficulty .................................................3  Cannot understand at all..................................... 4 |
|  | Child has difficulty being understood by parent/caretaker when speaks (Code 3,4) | No difficulty ........................................................1  Some difficulty.....................................................2  A lot of difficulty .................................................3  Cannot understand at all..................................... 4 |
| Learning | Compared with children of the same age, child has difficulty learning things (Code 3,4) | No difficulty ........................................................1  Some difficulty.....................................................2  A lot of difficulty .................................................3  Cannot learn things at all..................................... 4 |
| Playing | Compared with children of the same age, child has difficulty playing (Code 3,4) | No difficulty ........................................................1  Some difficulty.....................................................2  A lot of difficulty .................................................3  Cannot play at all................................................. 4 |
| Controlling  Behavior | Compared with children of the same age, how much child kick, bite or hit other children or adults (Code 5) | Not at all ...............................................................1  Less.......................................................................2  The same ..............................................................3  More.....................................................................4  A lot more ............................................................5 |
